# Clinical and Genetic Tumor Characteristics of Responding and Non-Responding Patients to PD-1 Inhibition in Hepatocellular Carcinoma

**DOI:** 10.3390/cancers12123830

**Published:** 2020-12-18

**Authors:** Stephan Spahn, Daniel Roessler, Radu Pompilia, Gisela Gabernet, Beryl Primrose Gladstone, Marius Horger, Saskia Biskup, Magdalena Feldhahn, Sven Nahnsen, Franz J. Hilke, Bernhard Scheiner, Jean-François Dufour, Enrico N. De Toni, Matthias Pinter, Nisar P. Malek, Michael Bitzer

**Affiliations:** 1 Department Internal Medicine I, Eberhard-Karls University, 72076 Tuebingen, Germany; stephan.spahn@med.uni-tuebingen.de (S.S.); Primrose.Beryl@med.uni-tuebingen.de (B.P.G.); Nisar.Malek@med.uni-tuebingen.de (N.P.M.); 2 Department of Medicine II, University Hospital Ludwig-Maximilians-University (LMU), 81377 Munich, Germany; Daniel.Roessler@med.uni-muenchen.de (D.R.); Enrico.deToni@med.uni-muenchen.de (E.N.D.T.); 3 Hepatology-Department of Biomedical Research, University of Bern, 3010 Bern, Switzerland; iuliana-pompilia.radu@insel.ch (R.P.); jean-francois.dufour@dbmr.unibe.ch (J.-F.D.); 4 University Clinic for Visceral Surgery and Medicine, Inselspital, University of Bern, 3010 Bern, Switzerland; 5 Quantitative Biology Center (QBiC), Eberhard-Karls University, 72076 Tuebingen, Germany; gisela.gabernet@qbic.uni-tuebingen.de (G.G.); sven.nahnsen@qbic.uni-tuebingen.de (S.N.); 6 Department of Diagnostic and Interventional Radiology, Eberhard-Karls University, 72076 Tuebingen, Germany; marius.horger@med.uni-tuebingen.de; 7 CeGaT GmbH and Praxis für Humangenetik, 72076 Tuebingen, Germany; Saskia.Biskup@humangenetik-tuebingen.de (S.B.); Magdalena.Feldhahn@cegat.de (M.F.); 8 Institute of Medical Genetics and Applied Genomics, Eberhard-Karls University, 72076 Tuebingen, Germany; Franz.hilke@charite.de; 9 Department of Dermatology, Venerology and Allergology, Charité–Universitätsmedizin Berlin, 10117 Berlin, Germany; 10 Division of Gastroenterology and Hepatology, Department of Internal Medicine III, Medical University of Vienna, 1090 Vienna, Austria; bernhard.scheiner@meduniwien.ac.at (B.S.); matthias.pinter@meduniwien.ac.at (M.P.); 11 Center for Personalized Medicine, Eberhard-Karls University, 72076 Tuebingen, Germany

**Keywords:** hepatocellular carcinoma, immunotherapy, biomarkers, microbiome, tumor mutational burden

## Abstract

**Simple Summary:**

Immune checkpoint inhibitors are dramatically reshaping cancer treatments in a multitude of advanced cancers, including hepatocellular carcinoma (HCC). However, only a subgroup of patients with HCC currently benefits from immunotherapy. Therefore, we performed a multicenter exploratory in-detail characterization of 99 patients with HCC and programmed cell death protein 1 (PD-1) blockade to identify markers associated with therapy response. Our study observed a better outcome for patients with low levels of alpha-fetoprotein, progression-free survival > 6 months, and relevant treatment responses in both Child–Pugh A and B patients. Genetic factors were analyzed in a subset of patients: Neither specific genetic patterns nor tumor mutational burden were associated with treatment response. Our cohort´s main finding suggests that the application of antibiotics 30 days before or after therapy initiation is associated with a worse outcome, indicating a possible influence of the host-microbiome modulation on the outcome of PD-1/PD-L1-targeted immunotherapy in HCC.

**Abstract:**

Immune checkpoint inhibitors (ICIs) belong to the therapeutic armamentarium in advanced hepatocellular carcinoma (HCC). However, only a minority of patients benefit from immunotherapy. Therefore, we aimed to identify indicators of therapy response. This multicenter analysis included 99 HCC patients. Progression-free (PFS) and overall survival (OS) were studied by Kaplan-Meier analyses for clinical parameters using weighted log-rank testing. Next-generation sequencing (NGS) was performed in a subset of 15 patients. The objective response (OR) rate was 19% median OS (mOS)16.7 months. Forty-one percent reached a PFS > 6 months; these patients had a significantly longer mOS (32.0 vs. 8.5 months). Child-Pugh (CP) A and B patients showed a mOS of 22.1 and 12.1 months, respectively. Ten of thirty CP-B patients reached PFS > 6 months, including 3 patients with an OR. Tumor mutational burden (TMB) could not predict responders. Of note, antibiotic treatment within 30 days around ICI initiation was associated with significantly shorter mOS (8.5 vs. 17.4 months). Taken together, this study shows favorable outcomes for OS with low AFP, OR, and PFS > 6 months. No specific genetic pattern, including TMB, could identify responders. Antibiotics around treatment initiation were associated with worse outcome, suggesting an influence of the host microbiome on therapy success.

## 1. Introduction

Hepatocellular carcinoma (HCC) is the most frequent primary liver cancer and, with nearly 800,000 patients dying annually of this disease, one of the most common cancer-related causes of death worldwide [1]. In advanced disease stages, several tyrosine kinase inhibitors (TKI) have been established as a systemic treatment option with sorafenib [2] and lenvatinib [3] as first-line treatment of HCC. For patients that have previously been treated with sorafenib, approval has also been granted to regorafenib [4], cabozantinib [5], and ramucirumab (if alpha fetoprotein level is ≥400 ng/mL) [6].

Immune checkpoint inhibitors (ICI) are dramatically reshaping cancer treatments in a multitude of advanced cancers, including hepatocellular carcinoma (HCC) [1,7,8,9]. Based on promising early and noncomparative clinical phase I/II trials, the Food and Drug Administration (FDA) has granted accelerated approval to the anti-PD-1-targeting antibodies nivolumab and pembrolizumab, and just recently for the combination of nivolumab and the anti-CTLA-4 antibody ipilimumab for patients that have been previously treated with sorafenib. Of 262 patients treated with nivolumab in the CheckMate-040 study, 20% reached either a complete (CR) or partial response (PR), 45% stable disease (SD), and 32% progressive disease (PD) as the best response [10]. Of note, the median duration of response was 17 months, indicating that some patients clearly benefit from this treatment [10]. The Keynote-224 study, in which 104 patients were treated with pembrolizumab, showed similar results with an objective response rate (ORR) of 17% [11]. The combination of nivolumab and ipilimumab was evaluated in 50 patients in the CheckMate-040 study, leading to an ORR of 30% with a median duration of response of 17.4 months [10]. The phase III IMbrave150 trial reported an improved progression-free (PFS) and overall survival (OS) for the combination of the anti-PD-L1 antibody atezolizumab and the anti-VEGF antibody bevacizumab compared to sorafenib and thus will become the new standard first-line therapy [12]. Nevertheless, all studies demonstrated that only a fraction of HCC patients benefit from PD-(L)1 inhibition. Yet, a peculiar effect of ICI, as opposed to that of TKIs, is the fact that patients showing an objective response exhibit a long-term benefit in terms of survival [9,13]. Unfortunately, the search for predictive clinical or molecular markers for ICI treatment in HCC has so far been disappointing. An exploratory analysis of PD-L1 expression could not discriminate between responders and non-responders [11]. Mismatch repair deficiency predicts response of solid tumors to programmed cell death protein 1 (PD-1) blockage [14]. However, only 2–3% of HCCs have been identified as microsatellite instability-high (MSI-high) tumors, which means that most of the HCC patients with an objective response do not necessarily have this kind of molecular alteration [14]. Data on the significance of tumor mutational burden (TMB), which was recently identified as a predictor of response to immunotherapy across multiple solid cancer types, are scarce in HCC [15]. 

Several specific mutated genes or somatic copy number alterations have been proposed to influence the response to ICI-treatment in different tumor entities [16]. In a small cohort of 27 HCC patients treated with ICI, tumor response correlated with activated WNT/β-catenin signaling [17]. All 10 patients in that study with CTNNB1 missense or in-frame mutations, or truncating mutations of AXIN1, showed tumor progression at the first interval scan. This observation is supported by preclinical evidence that β-catenin activation promotes immune escape and resistance to anti-PD-1 [18]. Despite these interesting initial results, genetic profiles of HCC are currently not routinely considered in clinical decision-making.

Besides tumor intrinsic genetic factors, manipulation of the gut microbiome by antibiotic treatment influenced the outcome of PD-1 blockade in mouse models and cancer patients [19]. Antibiotic therapy within the first administration of PD-1/PD-L1 antibodies was associated with worse progression-free (PFS) and overall survival (OS) of patients with lung or urogenital cancer [19]. Similar observations for HCC patients have not been reported yet.

Taken together, an unmet need for clinical and/or immunogenetic markers exists to predict response to ICI treatment in HCC. Here, we performed a multicenter exploratory in-detail characterization of patients with HCC and PD-1-blockade. 

## 2. Results

### 2.1. Description of the Cohort

Ninety-nine patients with advanced HCC were treated with anti-PD-1 agents at the four participating European centers during the study period and met the inclusion criteria. Of these, 67 patients received nivolumab and 32 patients received pembrolizumab. Patients´ demographics, baseline disease characteristics, and previous treatments are presented in Table 1. 

The median duration of follow-up was 16.7 months (interquartile range (IQR): 6.8–35.3 months). The median age was 69 years. Alcoholic liver cirrhosis was the most common underlying liver disease, followed by nonalcoholic steatohepatitis (NASH), and hepatitis B and C. Of the 99 patients, 32 (32%) had a reduced liver function at initiation of ICI treatment, as determined by Child–Pugh (CP) scores B or C. The study included 13 patients who received ICI as first-line therapy. The majority (*n* = 64 (65%)) received ICI as second-line therapy after sorafenib (62 patients) or lenvatinib (2 patients) treatment. Fifteen patients (15%) received ICI as third-line, five patients (5%) as fourth-line, and two patients (2%) as fifth-line therapy. Systemic therapies prior to initiation of ICI included TKIs as regorafenib or cabozantinib, but also experimental treatments (e.g., Notch inhibitor) or chemotherapy. 

### 2.2. Response to Treatment

**Figure 1:**
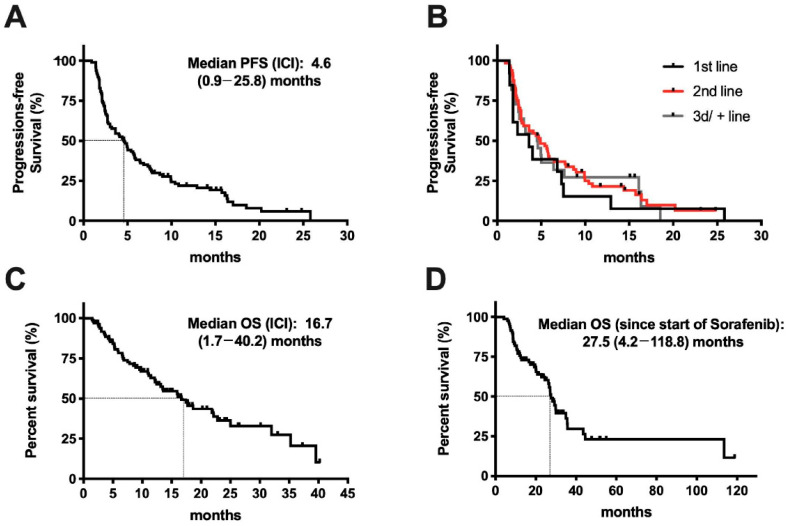
Response to immune checkpoint inhibitor (ICI) treatment in patients with advanced hepatocellular carcinoma (HCC). (**A**) Progression-free survival (PFS) and median PFS since start of ICI treatment in all 99 patients. (**B**) Comparison of PFS depending on the line of therapy (1st line: *n* = 13; 2nd line: *n* = 64; 3rd/+ line: *n* = 22). (**C**) Overall survival (OS) and median OS since start of ICI treatment in all 99 patients. (**D**) OS since start of therapy in the subgroup of patients with first line treatment of sorafenib (*n* = 83).

Four patients (4%) achieved a CR and 15 patients (15%) had a PR, resulting in an overall response rate (ORR) of 19%. The best treatment response was SD in 35 (35%) and PD in 45 (46%) patients. Median PFS (mPFS) during treatment with ICI was 4.6 months (range: 0.9–25.8+) with 13 patients still receiving therapy at the time of data cut-off (shown in Figure 1A). PFS did not significantly differ in patients receiving ICI as first (mPFS: 3.6 months), second (4.9 months, Hazard ratio (HR): 0.760, 95% confidence interval (CI) 0.406–1.423) or third/+ line (4.6 months; *p* = 0.7; HR: 0.827, 95% CI 0.400–1.710) (shown in Figure 1B). Median overall survival was 16.7 months (range: 1.7–40.2+) with 40 patients still alive at data cut-off (shown in Figure 1C). Of note, for the subgroup of patients with previous sorafenib treatment, the median overall survival since the start of sorafenib was 27.5 months (shown in Figure 1D).

**Figure 2:**
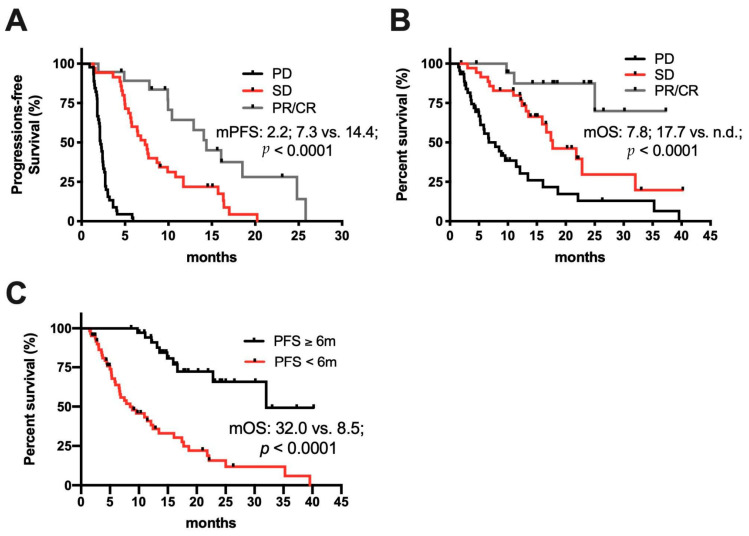
Association of best response to ICI treatment with progression-free and overall survival. (**A**) Comparison of PFS and (**B**) OS in patients with complete and partial response (CR/PR; *n* = 19) vs. stable disease (SD; *n* = 35) vs. progressive disease (PD; *n* = 45) as best treatment response to ICI. (**C**) Comparison of OS in patients with PFS ≥ 6 months (*n* = 41) vs. < 6 months (*n* = 58) since start of ICI treatment.

Next, we analyzed a possible association of an objective response (CR or PR) to PFS or OS. Patients with PD as the best response reached only a mPFS of 2.1 months. In contrast, patients with SD had a mPFS of 7.3 months (HR: 0.087, 95% CI 0.045–0.169) and those with an objective response (PR or CR) a mPFS of 14.4 months (*p* < 0.0001; HR: 0.030, 95% CI 0.013–0.074) (shown in Figure 2A). Similar results were obtained for median OS, with 7.8 months for patients with PD, 17.7 months for patients with SD (HR: 0.368, 95% CI 0.203–0.666), and “not reached” for patients with CR or PR (*p* < 0.0001; HR: 0.093, 95% CI 0.028–0.305) (shown in Figure 2B). Forty-one (41%) patients achieved a PFS exceeding 6 months. In these patients, OS was significantly longer as compared to patients with a PFS < 6 months (32.0 vs. 8.5 months; *p* < 0.0001; HR: 0.162, 95% CI 0.078–0.334) (shown in Figure 2C).

In summary, a subset of patients clearly benefits from ICI treatment, including prolonged disease stabilization and survival. Therefore, an exploratory in detail analysis of available clinical and molecular data was performed to identify potential parameters or conditions that might influence ICI treatment response in HCC.

### 2.3. Clinical Determinates of Response to ICI Treatment

**Figure 3:**
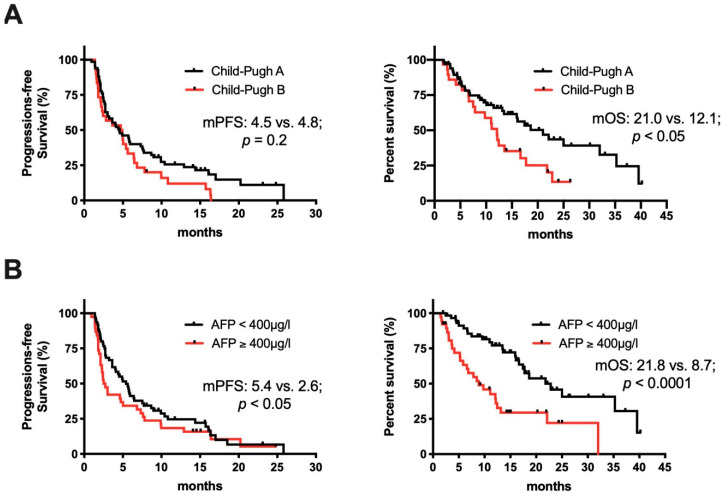
Clinical determinants of response to treatment with ICI. (**A**) Kaplan-Meier curve comparing progression-free survival (PFS) and overall survival (OS) of Child–Pugh A - (*n* = 67) vs. Child–Pugh B (*n* = 30) patients since start of ICI treatment. (**B**) Kaplan–Meier curve comparing patients with baseline AFP < 400 µg/L (*n* = 60) vs. ≥ 400 µg/L (*n* = 38).

Two important parameters that are routinely assessed for treatment decisions in HCC are the liver function, most often assessed by the Child–Pugh score, and serum AFP. In contrast to the phase I/II studies that led to FDA approval of nivolumab and pembrolizumab in HCC [10,20], nearly one third of the patients in our study had an impaired liver function, reflected by Child–Pugh B or even C. Ten (33%) of the 30 patients with Child–Pugh B showed a tumor control of more than 6 months, including three patients with PR. Additionally, the rate of PD as the best response was comparable for patients with Child–Pugh B (47%) compared to patients with preserved liver function (46%). (This group included patients with Child–Pugh A or without underlying liver cirrhosis: From now on combined and referred to as Child–Pugh A). Liver function did not affect mPFS and was similar in patients with Child–Pugh A compared to B (4.5 vs. 4.8 months; *p* = 0.25; HR 1.424, 95% CI 0.903–2.380). In patients with Child–Pugh B, mOS exceeded 12 months. Nevertheless, mOS was longer in patients with Child–Pugh A (22.1 vs. 12.1 months, respectively; *p* < 0.05; HR 2.088; 95% CI 1.185–3.677) (shown in Figure 3A). The two patients with Child–Pugh C liver function were excluded, as the small sample size did not allow for meaningful analysis.

AFP levels ≥ 400 µg/L are associated with decreased survival and are used as a biomarker to select HCC patients for treatment with the VEGFR2 inhibitor ramucirumab [6,21]. Thirty-eight patients had AFP levels ≥ 400 µg/L (median AFP in this group: 2198 µg/L; IQR: 798–11215 µg/L). Baseline levels of AFP < 400 µg/L (median AFP in this group: 16.5 µg/L; IQR: 5.3–66.1 µg/L) at the start of ICI treatment were associated with higher rates of PR or CR as best responses (24% vs. 13% in patients with AFP > 400 µg/L) and lower rates of PD (39% vs. 56%, respectively). Moreover, AFP < 400 µg/L was associated with a significantly longer median PFS (5.4 vs. 2.6 months; *p* < 0.05; HR 1.333, 95% CI 0.861–2.063) and OS (21.8 vs. 8.7 months; *p* < 0.0001; HR 2.819, 95% CI 1.560–4.967) (shown in Figure 3B).

Taken together, the analysis of these clinical parameters suggests that patients with both Child–Pugh A and B seem to benefit from ICI treatment, and that a previously described unfavorable outcome of patients with high AFP values at baseline is also true for patients undergoing treatment with ICI.

### 2.4. Molecular Determinates of Response to ICI Treatment

**Figure 4:**
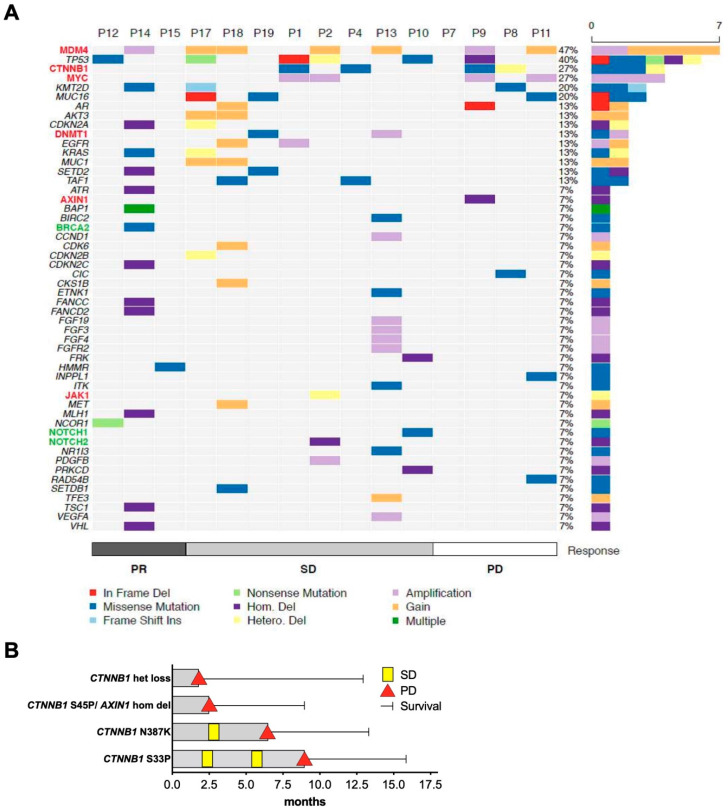
Genetic tumor alterations and treatment outcome. (**A**) Alterations in preselected genes of interest in patients with partial response (PR), stable disease (SD), and progressive disease (PD) as best treatment response. Within each of these three groups patients are sorted according to the duration of PFS (longer to shorter). Plotted frequencies include all alterations for each gene: In frame deletions (In Frame Del), missense mutations, frame shift insertions (Frame Shift Ins), homologous deletions (Hom. Del), amplifications, gain of copy number variations (gain), heterogeneous deletions (Hetero. Del), or multiple variations (Multiple). Selected genes that have been previously suggested to influence response to ICI are written in different colors: Green color indicates genes that have previously been associated with favorable outcomes, red color indicates genes with reported negative outcome to ICI treatment. (**B**) Swimmer plot illustrating the detailed clinical course of the 4 patients with WNT/β-catenin alterations. In the case of missense mutations, the introduced amino acid change is indicated.

Comprehensive genetic data obtained from HCC tissue samples were available in a subgroup of 15 patients. All tumor biopsies were performed before the initiation of ICI treatment. Figure 4A gives an overview of the detected genetic alterations in patients assorted to their best response with PR, SD, or PD. First, we compared the frequency of common oncogenic driver mutations in HCC to published data of the Cancer Genome Atlas (TCGA), and second, we explored possible associations with response or resistance to ICI treatment [22]. In line with the TCGA cohort, mutations in the tumor suppressor gene TP53 (in 40% of patients), the WNT-pathway oncogene CTNNB1 (27%), and amplifications in the oncogene MYC (27%) were among the most often altered genes (Figure 4A). In contrast to the TCGA cohort, no TERT promoter mutations were detected in our cohort. This is a consequence of the applied NGS gene panel, which did not cover the TERT promoter region. Moreover, whereas in the TCGA cohort only 9.7% of patients had MDM4 amplification, in our cohort gains and amplifications in MDM4 were found in 47% of patients.

Next, we analyzed whether alterations that have been previously linked to ICI response or resistance in HCC or other tumor entities were present in our cohort (shown in Figure 4A: Genes written in red have been associated with resistance and genes written in green with response to ICI treatment). Four patients were identified with alterations in the WNT/β-catenin pathway with three patients harboring activating mutations. Such alterations have been linked to resistance to ICI treatment in HCC, but only a few patients were reported so far with detailed information of the WNT/β-catenin alteration and the clinical outcome during treatment with ICI [17,18]. The clinical course of the 4 patients with WNT/β-catenin alterations is therefore shown in more detail in Figure 4B. Only one of the three patients with activating mutations showed a shorter PFS than the median PFS of the whole cohort. One patient with a mutation in CTNNB1 had SD for 9 months and another patient had SD for 6.2 months. This indicates that patients with alterations in the WNT/β-catenin pathway may still benefit from ICIs. 

Mutations in cancers other than HCC associated with a negative outcome for ICI treatment include inactivating mutations in JAK1/2 [23] or beta-2-microglobulin in melanoma [24], MDM4 amplifications, or EGFR alterations in different stage IV tumors [25] or STK11/LKB1 alterations in KRAS-mutant lung adenocarcinoma [26]. MDM4 gains or amplifications were detected in responding and non-responding patients in our cohort (shown in Figure 4A). One patient with MDM4 amplification even had PR as the best treatment response. EGFR gains or amplifications or a heterozygous loss of JAK1 were found in 3 patients that reached SD. Thus, negative predictive factors from other tumor entities could not identify patients with the worst outcome in our HCC cohort. 

Besides markers of resistance, specific further molecular alterations have been linked with a favorable response to ICI treatment. Examples are mutations in the DNA repair gene BRCA2 in melanoma [27] or in different stage IV cancers alterations in NOTCH, TERT, and NF1 [25]. Additionally, MYC amplifications were shown to cause a vulnerability to ICI treatment in an experimental setting [28]. In our cohort, the patient with a missense mutation in BRCA2 was one of the four patients with PR to ICI treatment; however, the functional relevance for this mutation has not been characterized yet. Four patients had MYC amplifications, but the median PFS tended to be shorter in patients with MYC amplifications with 5.1 vs. 6.5 months, suggesting that this alteration does not have a beneficial impact on the course under ICI treatment.

In summary, no single genetic alteration that has been previously suggested as a predictive marker was able to identify an outlier with a remarkably good tumor response or a rapid progression during ICI treatment in this subgroup of our HCC cohort, including patterns of WNT/β-catenin pathway activation.

### 2.5. Tumor Mutational Burden (TMB)

**Figure 5:**
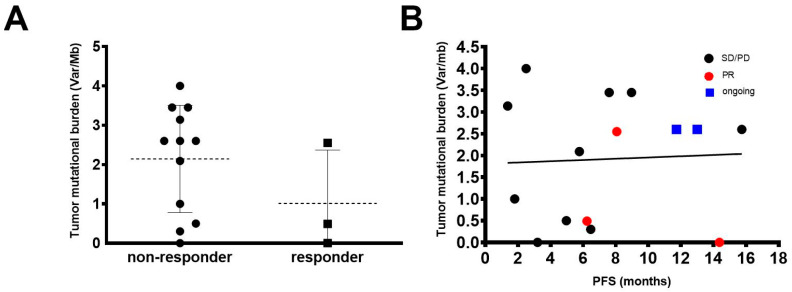
Treatment outcome and tumor mutational burden. (**A**) Comparison of tumor mutational burden (Var/Mb) of patients with an objective response (PR; responder; *n* = 3) and patients with SD or PD (non-responder; *n* = 12). (**B**) Correlation of tumor mutational burden with progression-free survival (PFS); Spearman r: 0.018.

No patient in our cohort had HCC with mismatch repair deficiency. We explored a potential correlation of TMB with response to ICI in HCC. The median TMB in our cohort was 2.6 Var/Mb (0–4 Var/Mb). Of note, the median TMB did not differ in responders and non-responders (1.0 vs. 2.1 Var/Mb; *p* ≥ 0.99) (shown in Figure 5A) with a tendency of lower TMB values in responding patients. Additionally, we did not find a correlation between TMB and PFS (Spearman r: 0.018) (shown in Figure 5B). While TMB may be a useful prognostic biomarker in other tumor types, in our cohort patients with PR could not be identified by this parameter.

### 2.6. Antibiotic Treatment and ICI Therapy

Modulation of the gut microbiome by antibiotic treatment has been associated with response to ICI treatment [19,29]. Therefore, we assessed patients’ survival according to whether they had received an antibiotic treatment 30 days or less prior to or after the initiation of ICI treatment. Of the 99 patients, 21 received an antibiotic treatment within this time frame. Thirteen patients received systemic antibiotics, which included broad spectrum cephalosporins or fluoroquinolones. In addition, 8 patients received the non-absorbable antibiotic rifaximin as a prophylaxis against hepatic encephalopathy. As a first step, we compared these 21 patients with the 78 patients without antibiotic treatment and found no difference in median PFS (5.0 vs. 7.6 months; *p* = 0.4; HR 1.198, 95% CI 0.709–2.022) or OS (12.1 vs. 17.4 months; *p* = 0.6; HR: 1.470, 95% CI 0.732–2.951) (shown in Figure S1A). However, looking at the patients with antibiotic treatment in more detail, there was a clear difference, with worse outcome of the 13 patients with a systemic antibiotic treatment compared to the 8 patients that received a prophylactic therapy of the non-absorbable drug rifaximin (shown in Figure S1B). 

**Figure 6:**
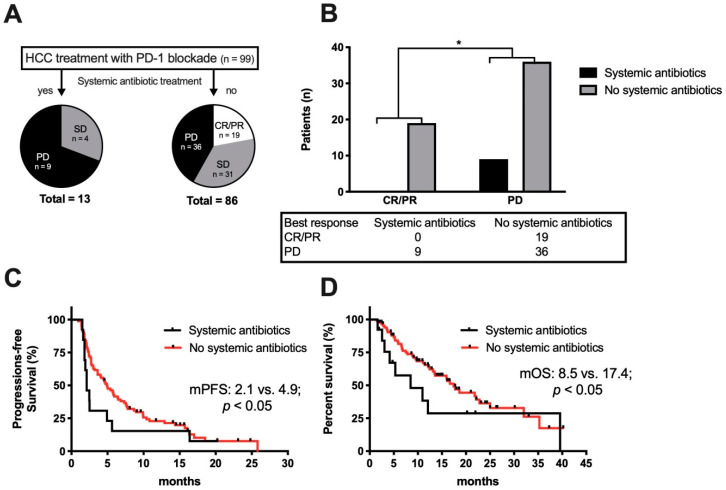
Influence of antibiotic treatment 30 days prior or after initiation of ICI treatment. (**A**) Flow chart demonstrating best treatment response in all ninety-nine patients depending on treatment with or without systemic antibiotics. (**B**) Comparison of the patient groups with an objective response to patients with progressive disease during anti-PD-1 and systemic antibiotic treatment. Patients with SD were not included in this analysis. The number of patients with CR/PR, or PD is indicated below the graph; * *p* < 0.05 using two-tailed chi-square. (**C**) Kaplan–Meier curve for progression-free survival (PFS) and (**D**) overall survival (OS) since start of ICI treatment in patients treated with systemically acting antibiotics ≤ 30 days prior or after initiation of ICI treatment (*n* = 13) compared to no systemic antibiotic treatment (*n* = 86).

Of note, all patients with an objective response to ICI treatment did not receive a systemic antibiotic treatment within the predefined time frame (shown in Figure 6A). A direct comparison of all patients with an objective response to all patients with PD as the best response demonstrated a significant association of systemic antibiotic treatment with PD (*p* < 0.05, two-tailed chi-square) (shown in Figure 6B). Furthermore, comparing patients with a systemically active antibiotic treatment to all other patients showed both a significantly shorter median PFS (2.1 vs. 4.9 months; *p* < 0.05; HR 1.65, 95% CI 0.904–3.000) (Figure 6C) and OS (8.5 vs. 17.4 months; *p* < 0.05 HR 1.757, 95% CI 0.844–3.660) (shown in Figure 6D). Taken together, our analysis revealed that systemic antibiotic treatment was associated with worse outcome in HCC patients undergoing anti-PD-1 treatment.

## 3. Discussion

This retrospective multicenter study found encouraging efficacy of PD-1 inhibition in a subset of HCC patients in a real-life setting. We therefore comprehensively explored possible clinical and molecular characteristics of responding and non-responding patients. The ORR and median PFS and OS were comparable to the results of published phase II studies testing nivolumab and pembrolizumab as second-line treatment in advanced HCC [10,11]. Of note, the observed median OS of 27.5 months since the start of sorafenib in sorafenib-pretreated patients underscores the progress made compared to the median OS with sorafenib alone of just 10.7 months in the SHARP trial more than ten years ago [2].

Despite promising results for some patients, our data highlights once again that in HCC, less than 20% of patients have objective responses to anti-PD-1-targeted monotherapy [10,11]. Among different tumor types, only a minority of patients treated with ICI derives a clinical benefit. Nevertheless, these patients seem to drive the increased survival observed in phase III studies [16]. A publication studying long-term survival to anti-PD-(L)1-therapy in a variety of cancer types showed that objective responses are in general limited to less than 30% of patients [30]. No HCC patients were included in that study, but Gauci et al. showed that patients responding at 3 months reached a median PFS of 30 months and 3- and 5-year survival rates of 84% and 64%, respectively. In our cohort, patients with objective responses had significant longer median PFS and OS as well. Additionally, patients who reached a PFS of at least 6 months derived a median OS, which was more than three times as long as for patients with a PFS below 6 months. Hence, our study once again supports the notion that an urgent need exists to identify prognostic biomarkers of responders and non-responders. This is especially crucial for HCC, as phase III studies of nivolumab as first-line or pembrolizumab as second-line treatment in advanced HCC did not reach the prespecified statistical primary endpoints despite remarkable data for median PFS and OS compared to sorafenib in the first-line or placebo in the second-line setting [6,13]. 

A notable clinical observation of better treatment response was an AFP baseline level < 400 µg/L, which most likely reflects poorer prognosis of HCC patients with high AFP levels in general [21]. Furthermore, the 30 patients in our cohort with reduced liver functions, determined by Child–Pugh status B, showed no difference in mPFS, but a decreased OS compared to Child–Pugh A patients. Yet, the median PFS of 4.8 and OS of 12.1 months of Child–Pugh B patients treated with anti-PD-1 antibodies were remarkable and exceeded so far the published data for this subgroup [31,32]. Observations that some patients with Child–Pugh B can substantially benefit from ICI treatment have been reported with median PFS and OS ranging between 1.6–4.6 and 5.9–8.6 months, respectively [31,33]. In a subgroup analysis of the CheckMate 040 study, 49 patients had Child–Pugh B [32]. In that study, PR was observed in 6 patients and SD in 21 patients, leading to a median PFS of 2.7 months and median OS of 7.6 months for the whole cohort. Looking at treatment-related adverse events and a subsequent need to discontinue the treatment in our study, 6 of the 30 (27%) patients with Child–Pugh B had to stop treatment. This is lower than the 39% described in the study from Kambhampati et al. but in the same range as reported by Kudo et al. [31,32]. Nevertheless, the available data of ICI treatment in patients with HCC and Child–Pugh B are comparable or even exceed previous reports with sorafenib as first-line treatment in Child–Pugh B patients from the observational studies GIDEON and INSIGHT with median OS of 5.2 months and 8.1 months, respectively [34,35]. Taking into consideration that CP-B patients may be especially vulnerable for variceal bleeding, a side effect of the combination therapy of atezolizumab plus bevacizumab [12]; our data support that ICI monotherapy warrants further investigation in Child–Pugh B patients for whom treatment options are still scare. 

Besides potential prognostic clinical characteristics, genetic and molecular biomarkers are a main focus of current research. The identification of molecular and genetic biomarker research in HCC is lagging behind other tumor types. We performed a detailed molecular analysis in a subgroup of patients; however, we could not attribute outliers in therapy response to one of the detected molecular alterations. Recent studies support the hypothesis that in the HCC WNT/β-catenin pathway, activation may lead to resistance to ICI treatment caused by immune evasion [17,18]. In line with these results, none of our patients with alteration in the WNT/β-catenin pathway had an objective response to treatment. Yet, in two of these three patients, the PFS and OS were similar or even exceeded the median of the whole cohort, suggesting that additional factors seem to contribute to the postulated immune evasion caused by WNT/β-catenin pathway activation. 

One of the major limitations of the exploratory analysis on genetic biomarkers in a subset of patients is the small sample size. Our results employing large NGS gene panel or exome analysis, however, indicate that translation of predictive biomarkers from other tumor types to HCC should be interpreted with caution. As an example, we found an objective response in a patient with an MDM4 alteration, which has been associated with hyper-progression in other tumor types or a possible lack of correlation between TMB and therapy response [25]. High TMB is hypothesized to increase the expression of immunogenic antigens followed by a boost of the anti-tumoral immune response and is regarded as a predictive biomarker for ICI response in a variety of cancers [15,16,36]. Yet, in our cohort, 58% of non-responders had a TMB which exceeded the highest TMB of a responder to ICI treatment, and statistically TMB did not differ in responding and non-responding patients. The median TMB of 2.6 Var/Mbp observed in our cohort was low when compared to other tumor entities that benefit from ICI treatment, especially melanoma, but was in a similar range in a recent study of 755 HCC cases in which the median TMB was 4 Var/Mbp and only 5% of patients had a TMB 10 Var/Mbp [36,37]. The results observed in our cohort suggest that TMB is not a useful predictive biomarker to identify ICI responders in HCC. 

The most remarkable finding of our study suggests a possible negative influence of systemic antibiotics in HCC when given within 30 days prior or after the initiation of ICI, as previously demonstrated for other tumor types [19,29]. Importantly, we did not only observe reduced PFS and OS in patients treated with systemic antibiotics within the initiation of ICI, but worse best responses by the modified Response Evaluation Criteria in Solid Tumors (mRECIST) as well. Taken together with our finding that usage of Rifaximin to treat hepatic encephalopathy, reflecting underlying liver disease, did not influence PFS and OS, these observations reassure that this novel finding in HCC is not a cofounder of underlying disease severity and propensity for the need for antibiotic treatment in these patients. In line with our observation, a small pilot study with fecal stool analysis of 8 patients with HCC and ICI treatment described that fecal samples from responding patients revealed higher taxa richness and more gene counts compared to non-responders [38]. In a dynamic analysis, that study found Proteobacteria increased from week 3 in non-responding patients, whereas responders showed the enrichment of several species, including *Akkermansia muciniphila* and *Ruminococcaceae* species. This is of interest because an increase in relative abundance of species affiliated with the Ruminococcus family has already been described in responding melanoma patients and a higher relative abundance of *Akkermansia muciniphila* has been determined in responding patients with renal cell carcinoma and non-small cell lung cancer [19,39]. The latter study could even show that in tumor mouse models an oral supplementation with *A. muciniphila* was able to restore the efficacy of the PD-1 blockade. In this context, it is important to mention that a substantial number of HCC patients receive the non-absorbable antibiotic rifaximin for the treatment or prevention of hepatic encephalopathy [40]. Despite its broad antibacterial action that covers Gram-positive, Gram-negative, aerobic, and anaerobic organisms [41], no differences could be detected for patients treated with rifaximin in respect to PFS or OS in our study. Of note, several investigations showed that rifaximin does not significantly change the overall microbiome composition [42,43,44], but Kakiyama et al. found a rifaximin-mediated reduction of the secondary to primary bile acid ratio, which was hypothesized to be a consequence of altered microbiome function [42]. In light of a recently published study, this finding might explain the missing influence of rifaximin treatment in our cohort. Ma et al. found that primary bile acids that originate from the gut lead to a selective accumulation of activated CXCR6+ natural killer T (NKT) cells that inhibit tumor growth in the liver [45]. Moreover, it was shown that an antibiotic treatment that reduced Gram-positive bacteria which convert primary to secondary bile acids induced inhibition of tumor growth. According to this study, the beneficial effect of rifaximin on the ratio of secondary to primary bile acids could explain the observed better outcome, compared with systemic broad-spectrum antibiotic treatment. Interestingly, a study demonstrated that an intratumoral microbiome in pancreatic cancer showed a higher alpha-diversity and specific microbiome signature associated with long-term survival [46]. Hence, another interesting hypothesis might be that an existing intra-tumoral microbiome in HCC is affected by systemic antibiotic treatment with an impact on immunotherapy responses. This interesting question should be addressed in future translational studies.

In summary, our in-depth exploratory analysis of patients with HCC and treatment with ICI revealed several noticeable results. Despite the application of a large NGS gene panel or exome sequencing, no reliable molecular determinant of therapy response or resistance could be attributed to the clinical outcome. Regardless of the small sample size and retrospective nature, this study suggests that ICI should be further investigated in patients with Child–Pugh B. Furthermore, treatment with antibiotics seems to negatively affect the response to ICI treatment in patients with advanced HCC. This important finding should be further investigated due to its high clinical relevance in larger patient cohorts.

## 4. Materials and Methods

### 4.1. Patients

All patients with HCC that presented to the University Hospital of Tuebingen (Germany), Munich (Germany), Vienna (Austria), and Bern (Switzerland) between August 2015 and December 2019 and who were treated with nivolumab or pembrolizumab were identified. Patients were included if they had undergone at least one follow-up examination or had been treated with ICI for at least one month. According to European guidelines, the diagnosis of HCC was either established histologically or radiologically [47]. The decision for systemic treatment was recommended by a multidisciplinary tumor board. Two of the 101 patients identified were excluded. The first patient had concurrent radiotherapy of spinal metastatic disease. The second was excluded due to prior liver transplantation. The retrospective analysis was approved by the local Ethic committees of Tubingen university (133/2018BO2), Munich university (18-488) and Vienna medical university (1759/2015 and 2033/2017). At Bern university all patients gave informed consent for scientific data analysis. The study analysis was conducted in accordance with the Declaration of Helsinki. 

### 4.2. Treatment and Response Monitoring

Nivolumab was given at a dose of 3 mg/kg every two weeks. Pembrolizumab was given at a fixed dose of 200 mg every three weeks. The electronic medical record was reviewed to extract clinical data on patients’ baseline characteristics, laboratory data including alpha-fetoprotein level (AFP), Child–Pugh score, extend of disease, etiology of HCC, history of prior treatments, dates, and numbers of systemic treatments. To assess treatment efficacy, CT or MRI scans were reviewed for complete (CR) and partial response (PR), stable disease (SD), or progressive disease (PD) by an experienced radiologist according to the modified Response Evaluation Criteria in Solid Tumors (mRECIST) [46]. Staging examinations were performed every 4–12 weeks. Progression-free survival (PFS) and overall survival (OS) were calculated beginning from the first treatment of nivolumab or pembrolizumab. Patients who did not show progression during the observation period were censored at the last imaging examination. For survival analysis, seven patients had to be censored at the date of last contact due to lost to follow up. Patients who were alive at the data cut-off date were censored at the date of last contact. 

### 4.3. Genomic Analyses of Tumor/Normal Tissue 

Tumor and normal tissue were genetically characterized either by next generation panel sequencing or whole-exome sequencing (WES) for a subgroup of 15 patients. The only selection criteria for genetic analyses was the availability of tumor specimen, as no additional biopsies were performed for this study. DNA for panel sequencing was extracted from Formalin-Fixed Paraffin-Embedded (FFPE)-embedded tissues that were treated according to standard pathology laboratory procedures. DNA for exome sequencing was extracted from fresh frozen tissue sections. Pathology procedures included the validation of the tumor type, determination of the percentage of tumor cells in the section and microdissection of tumor areas. Panel sequencing for 12 patients was performed by CeGaT GmbH, Tübingen or the Institute of Medical Genetics and Applied Genomics, Tübingen. During the study period, the NGS panel was expanded from 649 to 710 genes (an overview of the applied panel versions is given in Table S1). Reported results included somatic single nucleotide variants (SNVs), small insertions and deletions (INDELs), selected fusions, estimated tumor mutational burden, and microsatellite instability analysis. Tumor and normal DNA of 3 additional patients were sequenced exome-wide at the Institute of Medical Genetics and Applied Genomics. The somatic variants from all patients were aggregated in a MAF file and analyzed in R (v 3.6.1) with the Bioconductor Maftools package v2.0.10 [48]. Tumor mutational burden is defined as the number of somatic SNV-, InDel-, and essential splice site variants (NAF ≥ 0.1) per mega base of coding DNA. Truncating variants in tumor suppressor genes and known driver mutations as well as somatic variants with an in-house frequency of ≥ 1% were not accounted for. 

### 4.4. Statistical Analysis 

Associations between clinical characteristics and PFS or OS were analyzed with the Kaplan-Meier method and compared by weighted log-rank testing using a generalized Fleming–Harrington test with more weight given to earlier failures to emphasize early differences and taking censoring at later time points into account. Hazard ratios (HR) are reported with the 95% confidence interval (95% CI). To analyze the impact of tumor mutational burden unpaired, a two-tailed Student’s *t*-test was used. For correlation studies, Spearman r was determined. A two-tailed chi-square was used to analyze the association of treatment with systemic antibiotics with best radiological responses. *p*-values < 0.05 were accepted to indicate statistical significance. Data were analyzed using Graph Pad version 8 (GraphPad Software, San Diego, CA, USA) and Stata/SE (15.1 version; College Station, TX, USA). 

## 5. Conclusions

This explorative, hypothesis-generating study observed a better outcome to ICI treatment for patients with low AFP levels, relevant treatment responses in both Child–Pugh A and B patients, and no association of TMB with treatment response. The application of systemic antibiotics within therapy initiation was associated with a worse outcome, a finding that should be addressed in future translational studies.

## Figures and Tables

**Table 1:** Baseline characteristics of 99 eligible patients treated with programmed cell death protein 1 (PD-1)-targeted immunotherapy from August 2015 to December 2019.

Patients	*No* = 99
Age—Year (Median)	69.0 (20.8–87.0)
Liver disease—no (%)	
Hepatitis B	13 (13%)
Hepatitis C	24 (24%)
Alcohol	32 (32%)
NASH	16 (16%)
Budd–Chiari syndrome	2 (2%)
Haemochromatosis	3 (3%)
Unknown hepatopathy	9 (9%)
HCC without cirrhosis	15 (15%)
Extrahepatic spread—no (%)	54 (55%)
Macrovascular invasion—no (%)	24 (24%)
Biochemical analysis at baseline (median)	
Alpha-fetoprotein µg/L (*n* = 98 ^†^)	90.5 (0.7–60500)
Child–Pugh class at baseline	
A	67 (68%)
B	30 (30%)
B7	14 (14%)
B8	11 (11%)
B9	3 (3%)
C	2 (2%)
Previous locoregional treatments—no (%)	
Surgical resection	31 (31%)
Radiofrequency ablation	22 (23%)
Percutaneous Ethanol Injection	1 (1%)
TACE	32 (32%)
SIRT	14 (14%)
Line of treatment ICI was used—no (%)	
1st line	13 (13%)
2nd line	64 (65%)
3rd line	15 (15%)
4th line	5 (5%)
5th line	2 (2%)
Previous systemic treatments—no (%)	
Sorafenib (1st line)	84 (85%)
Length of sorafenib treatment—months (median)	3.1 (0.3–32)
Lenvatinib	2 (2%)
FOLFOX/XELOX	3 (3%)
Cabozantinib	2 (2%)
Regorafenib	16 (16%)
Experimental drug	6 (6%)
Best treatment response to PD-1 Inhibitor—no (%)	
Complete response	4 (4%)
Partial response	15 (15%)
Stable disease	35 (35%)
Progressive disease	45 (45%)

No: Number, (values): Range, †: For one patient no value at baseline available, TACE: Transarterial chemoembolization, SIRT: Selective internal radiotherapy.

## Supplementary Materials

The following are available online at https://www.mdpi.com/2072-6694/12/12/3830/s1, Figure S1: Influence of antibiotic treatment prior or after initiation of ICI treatment. Table S1: Genes represented in different panel versions.
