# Supplementary material for: Clinical and Genetic Tumor Characteristics of Responding and Non-Responding Patients to PD-1 Inhibition in Hepatocellular Carcinoma
Source: Cancers (Basel). 2020 Dec 18;12(12):3830. doi: 10.3390/cancers12123830 (PMC7766321; doi:10.3390/cancers12123830)
Supplement: Supplementary file 1 [file cancers-12-03830-s001.zip › supp/Supplementary_Table 1.pdf]

Supplementary Table 1. *Genes represented in different panel versions*

	Version V3 (649 Gene)	Version V4 (710 Gene)	Version V3 (678 Gene)
ABCB1	ABCB1		ABCB1
ABCC2	ABCC2		ABCC2
ABCC4			ABCC4
ABCG2	ABCG2		ABCG2
ABL1	ABL1	ABL1	ABL1
ABL2	ABL2	ABL2	ABL2
ACD	ACD	ACD	
ACE			
ACO1			ACO1
ACTB			ACTB
ACVR1	ACVR1		
ACVR1B	ACVR1B		ACVR1B
ACVR2A			ACVR2A
ADAM10			ADAM10
ADAMTS18			
ADCY1			ADCY1
ADGRA2			ADGRA2
ADH1A			
ADH1B			
ADH1C			
ADRB1			
ADRB2			
AHR			
AIP		AIP	
AJUBA	AJUBA	AJUBA	AJUBA
AKAP9			AKAP9
AKT1	AKT1	AKT1	AKT1
AKT2	AKT2	AKT2	AKT2
AKT3	AKT3	AKT3	AKT3
ALDH1A1			
ALK	ALK	ALK	ALK
ALOX12B			ALOX12B
ALOX5			
AMER1	AMER1	AMER1	AMER1
ANK3			ANK3
ANKRD26		ANKRD26	
APAF1			APAF1
APC	APC	APC	APC
APCDD1			APCDD1
AR	AR	AR	AR
ARAF	ARAF	ARAF	ARAF
ARFRP1	ARFRP1		ARFRP1
ARHGAP35	ARHGAP35	ARHGAP35	ARHGAP35
ARHGEF6			ARHGEF6
ARID1A	ARID1A	ARID1A	ARID1A
ARID1B	ARID1B	ARID1B	ARID1B
ARID2	ARID2	ARID2	ARID2

ARID5B	ARID5B	ARID5B	ARID5B
ARNT	ARNT		
ASXL1	ASXL1	ASXL1	ASXL1
ASXL2		ASXL2	ASXL2
ATF1	ATF1		
ATG2B		ATG2B	
ATM	ATM	ATM	ATM
ATP1A1	ATP1A1	ATP1A1	ATP1A1
ATP5B	ATP5B		
ATR	ATR	ATR	ATR
ATRX	ATRX	ATRX	ATRX
AURKA	AURKA	AURKA	AURKA
AURKB	AURKB	AURKB	AURKB
AURKC	AURKC	AURKC	
AXIN1	AXIN1	AXIN1	AXIN1
AXIN2	AXIN2	AXIN2	AXIN2
AXL	AXL	AXL	AXL
AZGP1	AZGP1		
B2M	B2M	B2M	B2M
BACH1			BACH1
BAP1	BAP1	BAP1	BAP1
BARD1	BARD1	BARD1	BARD1
BBC3			BBC3
BCL10	BCL10	BCL10	
BCL11A	BCL11A	BCL11A	
BCL11B	BCL11B	BCL11B	
BCL2	BCL2	BCL2	BCL2
BCL2A1			
BCL2L1	BCL2L1		BCL2L1
BCL2L11			BCL2L11
BCL2L2	BCL2L2		BCL2L2
BCL3	BCL3	BCL3	
BCL6	BCL6	BCL6	BCL6
BCL9	BCL9	BCL9	
BCLAF1			BCLAF1
BCOR	BCOR	BCOR	BCOR
BCORL1	BCORL1	BCORL1	BCORL1
BCR	BCR	BCR	BCR
BIRC2	BIRC2	BIRC2	
BIRC3	BIRC3	BIRC3	
BIRC5	BIRC5	BIRC5	
BLM	BLM	BLM	BLM
BMPR1A	BMPR1A	BMPR1A	BMPR1A
BRAF	BRAF	BRAF	BRAF
BRCA1	BRCA1	BRCA1	BRCA1
BRCA2	BRCA2	BRCA2	BRCA2
BRD3		BRD3	
BRD4	BRD4	BRD4	BRD4
BRE	BRE		
BRIP1	BRIP1	BRIP1	BRIP1

BTG1			BTG1
BTK	BTK	BTK	BTK
BTNL2	BTNL2	BTNL2	
BUB1B	BUB1B	BUB1B	BUB1B
C11ORF30	C11ORF30	C11ORF30	CAD
CALR		CALR	
CAMK2G		CAMK2G	
CARD11	CARD11	CARD11	CARD11
CARM1			CARM1
CASP8	CASP8	CASP8	CASP8
CAST			CAST
CBFB	CBFB	CBFB	CBFB
CBL	CBL	CBL	CBL
CBLB		CBLB	CBLB
CBLC		CBLC	CBLC
CCAR1			CCAR1
CCDC6	CCDC6	CCDC6	
CCND1	CCND1	CCND1	CCND1
CCND2	CCND2	CCND2	CCND2
CCND3	CCND3	CCND3	CCND3
CCNE1	CCNE1	CCNE1	CCNE1
CD1D	CD1D		CD1D
CD274	CD274	CD274	CD274
CD276			CD276
CD38		CD38	
CD52		CD52	
CD58		CD58	
CD70	CD70		CD70
CD79A	CD79A	CD79A	CD79A
CD79B	CD79B	CD79B	CD79B
CD82	CD82	CD82	
CDC27	CDC27		CDC27
CDC73	CDC73	CDC73	CDC73
CDH1	CDH1	CDH1	CDH1
CDH2	CDH2	CDH2	
CDH11		CDH11	
CDH20			CDH20
CDH5			
CDK12	CDK12	CDK12	CDK12
CDK2			CDK2
CDK4	CDK4	CDK4	CDK4
CDK6	CDK6	CDK6	CDK6
CDK8	CDK8	CDK8	CDK8
CDKN1A	CDKN1A	CDKN1A	CDKN1A
CDKN1B	CDKN1B	CDKN1B	CDKN1B
CDKN1C		CDKN1C	
CDKN2A	CDKN2A	CDKN2A	CDKN2A
CDKN2B	CDKN2B	CDKN2B	CDKN2B
CDKN2C	CDKN2C	CDKN2C	CDKN2C
CDX2	CDX2		

CEBPA	CEBPA	CEBPA	CEBPA
CEP57	CEP57	CEP57	
CHD1		CHD1	
CHD2	CHD2	CHD2	
CHD3			CHD3
CHD4	CHD4	CHD4	CHD4
CHD8			CHD8
CHEK1	CHEK1	CHEK1	CHEK1
CHEK2	CHEK2	CHEK2	CHEK2
CHUK			CHUK
CIC	CIC	CIC	CIC
CIITA		CIITA	
CKS1B	CKS1B	CKS1B	
CLTC			CLTC
CNOT1			CNOT1
CNOT3		CNOT3	
CNTNAP1			CNTNAP1
COL1A1	COL1A1	COL1A1	COL1A1
COL3A1			COL3A1
COMMD1		COMMD1	
COMT			COMT
CRBN			CRBN
CREB1	CREB1	CREB1	CREB1
CREBBP	CREBBP	CREBBP	CREBBP
CRKL	CRKL	CRKL	CRKL
CRLF2			CRLF2
CRTC1	CRTC1	CRTC1	CRTC1
CRTC2		CRTC2	
CRTC3			CRTC3
CSDE1			CSDE1
CSF1R	CSF1R	CSF1R	CSF1R
CSF2		CSF2	
CSF3R		CSF3R	
CSMD1		CSMD1	
CSNK1A1		CSNK1A1	
CTCF	CTCF	CTCF	CTCF
CTLA4		CTLA4	CTLA4
CTNNA1	CTNNA1	CTNNA1	CTNNA1
CTNNB1	CTNNB1	CTNNB1	CTNNB1
CTNND1			CTNND1
CTTN			CTTN
CUL1			CUL1
CUL3	CUL3		
CUL4A			CUL4A
CUL4B	CUL4B	CUL4B	CUL4B
CUX1	CUX1	CUX1	CUX1
CXCR4		CXCR4	
CYLD	CYLD	CYLD	CYLD
CYP1A2	CYP1A1		
CYP1A2	CYP1A2		

CYP17A1			CYP17A1
CYP1B1			CYP1B1
CYP2A6	CYP2A6		
CYP2A7		CYP2A7	
CYP2B6	CYP2B6		
CYP2C19	CYP2C19		CYP2C19
CYP2C8	CYP2C8		CYP2C8
CYP2C9	CYP2C9		CYP2C9
CYP2D6	CYP2D6		CYP2D6
CYP2E1	CYP2E1		
CYP2J2			
CYP3A4	CYP3A4		CYP3A4
CYP3A5	CYP3A5		CYP3A5
DAXX	DAXX	DAXX	DAXX
DCC	DCC	DCC	
DCUN1D1			DCUN1D1
DDB2	DDB2	DDB2	DDB2
DDIT3	DDIT3		
DDR1		DDR1	DDR1
DDR2	DDR2	DDR2	DDR2
DDX11		DDX11	
DDX3X	DDX3X	DDX3X	DDX3X
DDX41		DDX41	
DDX5			DDX5
DEK	DEK	DEK	
DHFR		DHFR	
DIAPH1	DIAPH1		DIAPH1
DICER1	DICER1	DICER1	DICER1
DIDO1			DIDO1
DIS3	DIS3	DIS3	DIS3
DIS3L2		DIS3L2	
DKC1		DKC1	
DLL1			
DLL3			
DLL4			
DMD			DMD
DNMT1	DNMT1	DNMT1	DNMT1
DNMT3A	DNMT3A	DNMT3A	DNMT3A
DNMT3B			DNMT3B
DOT1L	DOT1L	DOT1L	DOT1L
DPYD	DPYD	DPYD	DPYD
DRD2			
DST	DST		
E2F3			E2F3
EBP		EBP	
EED			EED
EEF1A1			EEF1A1
EGFL7			EGFL7
EGFR	EGFR	EGFR	EGFR
EGLN1		EGLN1	

EGR2		EGR2	
EGR3	EGR3	EGR3	EGR3
EIF1AX			EIF1AX
EIF4A2			EIF4A2
ELAC2	ELAC2	ELAC2	
ELANE		ELANE	
ELF3	ELF3	ELF3	ELF3
EML4	EML4	EML4	EML4
ENG	ENG		
EP300	EP300	EP300	EP300
EPAS1		EPAS1	
EPCAM	EPCAM	EPCAM	EPCAM
EPHA2	EPHA2	EPHA2	EPHA2
EPHA3	EPHA3	EPHA3	EPHA3
EPHA4		EPHA4	
EPHA5	EPHA5		EPHA5
EPHA6			EPHA6
EPHA7	EPHA7		EPHA7
EPHB1	EPHB1		EPHB1
EPHB2			EPHB2
EPHB4	EPHB4	EPHB4	
EPHB6	EPHB6	EPHB6	EPHB6
EPHX1	EPHX1		
EPPK1			EPPK1
ERBB2	ERBB2	ERBB2	ERBB2
ERBB3	ERBB3	ERBB3	ERBB3
ERBB4	ERBB4	ERBB4	ERBB4
ERCC1	ERCC1	ERCC1	ERCC1
ERCC2	ERCC2	ERCC2	ERCC2
ERCC3	ERCC3	ERCC3	ERCC3
ERCC4	ERCC4	ERCC4	ERCC4
ERCC5	ERCC5	ERCC5	ERCC5
ERG	ERG	ERG	ERG
ERRFI1	ERRFI1	ERRFI1	
ESR1	ESR1	ESR1	ESR1
ESR2		ESR2	ESR2
ETNK1		ETNK1	
ETS1	ETS1	ETS1	
ETV1	ETV1	ETV1	ETV1
ETV4	ETV4	ETV4	ETV4
ETV5	ETV5	ETV5	ETV5
ETV6	ETV6	ETV6	ETV6
EWSR1	EWSR1	EWSR1	EWSR1
EXO1		EXO1	
EXT1	EXT1	EXT1	EXT1
EXT2	EXT2	EXT2	EXT2
EZH1	EZH1	EZH1	EZH1
EZH2	EZH2	EZH2	EZH2
F5			
FAM175A	FAM175A	FAM175A	FAM175A

FAM46C	FAM46C	FAM46C	FAM46C
FAN1		FAN1	
FANCA	FANCA	FANCA	FANCA
FANCB	FANCB	FANCB	
FANCC	FANCC	FANCC	FANCC
FANCD2	FANCD2	FANCD2	FANCD2
FANCE	FANCE	FANCE	FANCE
FANCF	FANCF	FANCF	FANCF
FANCG	FANCG	FANCG	FANCG
FANCI	FANCI	FANCI	FANCI
FANCL	FANCL	FANCL	FANCL
FANCM	FANCM	FANCM	FANCM
FAS	FAS	FAS	FAS
FAT1	FAT1	FAT1	FAT1
FAT3			FAT3
FBN1			FBN1
FBXO11			FBXO11
FBXW7	FBXW7	FBXW7	FBXW7
FES	FES	FES	FES
FGF10	FGF10	FGF10	FGF10
FGF12			FGF12
FGF14	FGF14	FGF14	FGF14
FGF19	FGF19	FGF19	FGF19
FGF2		FGF2	
FGF23	FGF23	FGF23	FGF23
FGF3	FGF3	FGF3	FGF3
FGF4	FGF4	FGF4	FGF4
FGF5		FGF5	
FGF6	FGF6	FGF6	FGF6
FGF7			FGF7
FGFBP1	FGFBP1	FGFBP1	FGFBP1
FGFR1	FGFR1	FGFR1	FGFR1
FGFR2	FGFR2	FGFR2	FGFR2
FGFR3	FGFR3	FGFR3	FGFR3
FGFR4	FGFR4	FGFR4	FGFR4
FH	FH	FH	FH
FKBP1A		FKBP1A	
FLCN	FLCN	FLCN	FLCN
FLI1	FLI1	FLI1	
FLT1	FLT1	FLT1	FLT1
FLT3			FLT3
FLT4	FLT4	FLT4	FLT4
FN1	FN1		FN1
FOXA1	FOXA1	FOXA1	FOXA1
FOXA2	FOXA2	FOXA2	FOXA2
FOXE1	FOXE1	FOXE1	FOXE1
FOXL2	FOXL2	FOXL2	FOXL2
FOXO1	FOXO1	FOXO1	
FOXO3	FOXO3	FOXO3	
FOXP1	FOXP1	FOXP1	FOXP1

FOXP4			
FOXQ1	FOXQ1	FOXQ1	FOXQ1
FRK		FRK	
FRS2	FRS2	FRS2	
FUBP1	FUBP1	FUBP1	FUBP1
FUS	FUS	FUS	
FYN		FYN	
G6PD	G6PD	G6PD	
GABRA6	GABRA6	GABRA6	GABRA6
GALNT12	GALNT12	GALNT12	
GATA1	GATA1	GATA1	GATA1
GATA2	GATA2	GATA2	GATA2
GATA3	GATA3	GATA3	GATA3
GATA4	GATA4	GATA4	
GATA6	GATA6	GATA6	
GDNF	GDNF		
GID4	GID4		GID4
GLA			GLA
GLDN		GLDN	
GLI1	GLI1	GLI1	
GLI2		GLI2	
GNA11	GNA11	GNA11	GNA11
GNA13	GNA13	GNA13	GNA13
GNAI1			GNAI1
GNAQ	GNAQ	GNAQ	GNAQ
GNAS	GNAS	GNAS	GNAS
GOLGA5			GOLGA5
GOT1	GOT1		GOT1
GPC3	GPC3	GPC3	
GPER1		GPER1	
GPR124	GPR124	GPR124	
GPS2			GPS2
GPX1			GPX1
GREM1		GREM1	GREM1
GRIN2A	GRIN2A	GRIN2A	GRIN2A
GRM3	GRM3	GRM3	GRM3
GSK3A		GSK3A	
GSK3B	GSK3B		GSK3B
GSTM1	GSTM1		
GSTP1	GSTP1		GSTP1
GSTT1	GSTT1		
GUCY1A2			GUCY1A2
GUSB	GUSB		GUSB
H3F3A	H3F3A	H3F3A	H3F3A
H3F3B	H3F3B		
H3F3C			H3F3C
HCFC1			HCFC1
HCK		HCK	
HERC1			HERC1
HGF	HGF	HGF	HGF

HIF1A	HIF1A	HIF1A	HIF1A
HIST1H1C			HIST1H1C
HIST1H2BD			HIST1H2BD
HIST1H3B	HIST1H3B	HIST1H3B	HIST1H3B
HLA-A	HLA-A	HLA-A	HLA-A
HLA-B	HLA-B	HLA-B	HLA-B
HLA-C	HLA-C	HLA-C	
HLA-DPA1		HLA-DPA1	
HLA-DPB1		HLA-DPB1	
HLA-DQA1		HLA-DQA1	
HLA-DQB1		HLA-DQB1	
HLA-DRA		HLA-DRA	
HLA-DRB1		HLA-DRB1	
HLF	HLF	HLF	
HMGA2	HMGA2	HMGA2	
HMGCR			
HMGN1		HMGN1	
HMOX2		HMOX2	
HNF1A	HNF1A	HNF1A	HNF1A
HNF1B	HNF1B	HNF1B	
HOXA3			
HOXA9	HOXA9		
HOXB13	HOXB13	HOXB13	
HOXD8		HOXD8	
HRAS	HRAS	HRAS	HRAS
HSD3B1	HSD3B1	HSD3B1	HSD3B1
HSP90AA1	HSP90AA1	HSP90AA1	
HSP90AB1	HSP90AB1	HSP90AB1	HSP90AB1
HSPA8			HSPA8
ICOSLG			ICOSLG
ID3		ID3	
IDH1	IDH1	IDH1	IDH1
IDH2	IDH2	IDH2	IDH2
IFNGR1		IFNGR1	IFNGR1
IFNGR2		IFNGR2	
IGF1			IGF1
IGF1R	IGF1R	IGF1R	IGF1R
IGF2	IGF2	IGF2	IGF2
IGF2R	IGF2R	IGF2R	IGF2R
IKBKB	IKBKB	IKBKB	
IKBKE	IKBKE	IKBKE	IKBKE
IKZF1	IKZF1	IKZF1	IKZF1
IKZF3		IKZF3	
IL10			IL10
IL1B		IL1B	
IL1RN		IL1RN	
IL2	IL2	IL2	
IL21R	IL21R	IL21R	
IL6		IL6	
IL6ST	IL6ST	IL6ST	

IL7R	IL7R	IL7R	IL7R
ING1	ING1		ING1
ING4	ING4	ING4	
INHBA	INHBA		INHBA
INPP4B	INPP4B	INPP4B	INPP4B
INPPL1	INPPL1	INPPL1	INPPL1
INSR			INSR
IRF1		IRF1	
IRF2	IRF2		
IRF4	IRF4		IRF4
IRF6	IRF6		
IRS1			IRS1
IRS2	IRS2	IRS2	IRS2
IRS4			
ITGB2	ITGB2		
ITK	ITK	ITK	
JAG1			JAG1
JAG2			JAG2
JAK1	JAK1	JAK1	JAK1
JAK2	JAK2	JAK2	JAK2
JAK3	JAK3	JAK3	JAK3
JUN	JUN	JUN	JUN
KALRN			KALRN
KAT6A	KAT6A	KAT6A	KAT6A
KCNH2			KCNH2
KCNJ11			
KCNJ5	KCNJ5		
KCNQ1			KCNQ1
KDM5A	KDM5A	KDM5A	KDM5A
KDM5C	KDM5C	KDM5C	KDM5C
KDM6A	KDM6A	KDM6A	KDM6A
KDR	KDR	KDR	KDR
KEAP1	KEAP1	KEAP1	KEAP1
KEL	KEL		KEL
KIAA1549	KIAA1549	KIAA1549	
KIT	KIT	KIT	KIT
KLF2		KLF2	
KLF4	KLF4	KLF4	KLF4
KLF5			KLF5
KLF6	KLF6		
KLHDC8B		KLHDC8B	
KLHL6	KLHL6	KLHL6	KLHL6
KMT2A	KMT2A	KMT2A	KMT2A
KMT2B	KMT2B	KMT2B	KMT2B
KMT2C	KMT2C	KMT2C	KMT2C
KMT2D	KMT2D	KMT2D	KMT2D
KRAS	KRAS	KRAS	KRAS
LAMA2			LAMA2
LAMP1	LAMP1		
LATS1	LATS1	LATS1	LATS1

LATS2	LATS2	LATS2	LATS2
LCK	LCK	LCK	
LCP1			LCP1
LDLR			LDLR
LGI1	LGI1		
LIFR	LIFR		LIFR
LIG4	LIG4	LIG4	
LIMK2		LIMK2	
LMNA			LMNA
LMO1	LMO1	LMO1	LMO1
LPP	LPP		LPP
LRP1B	LRP1B	LRP1B	LRP1B
LRP6			
LRRK2	LRRK2	LRRK2	LRRK2
LTK	LTK	LTK	
LYL1	LYL1		
LYN	LYN	LYN	LYN
LZTR1	LZTR1	LZTR1	LZTR1
MAD2L2		MAD2L2	
MAF	MAF		
MAFB	MAFB	MAFB	
MAGEA1	MAGEA1	MAGEA1	
MAGI1	MAGI2	MAGI1	
MAGI2		MAGI2	
MALAT1			MALAT1
MALT1	MALT1		
MAML1	MAML1	MAML1	
MAML2			MAML2
MAP2K1	MAP2K1	MAP2K1	MAP2K1
MAP2K2	MAP2K2	MAP2K2	MAP2K2
MAP2K3		MAP2K3	
MAP2K4	MAP2K4	MAP2K4	MAP2K4
MAP2K5		MAP2K5	
MAP2K6		MAP2K6	
MAP2K7		MAP2K7	MAP2K7
MAP3K1	MAP3K1	MAP3K1	MAP3K1
MAP3K13			MAP3K13
MAP3K14		MAP3K14	
MAP3K3		MAP3K3	MAP3K3
MAP3K4		MAP3K4	MAP3K4
MAP3K6	MAP3K6	MAP3K6	
MAP4K1			MAP4K1
MAP4K3			MAP4K3
MAPK1	MAPK1	MAPK1	MAPK1
MAPK11		MAPK11	
MAPK12		MAPK12	
MAPK3		MAPK3	
MAPK8	MAPK8		
MAPK8IP1	MAPK8IP1	MAPK8IP1	MAPK8IP1
MAX	MAX	MAX	MAX

MBD1	MBD1	MBD1	MBD1
MC1R	MC1R	MC1R	MC1R
MCL1	MCL1	MCL1	MCL1
MDC1		MDC1	MDC1
MDM2	MDM2	MDM2	MDM2
MDM4	MDM4	MDM4	MDM4
MECOM	MECOM	MECOM	MECOM
MED1			MED1
MED12	MED12	MED12	MED12
MED17			MED17
MED23			MED23
MEF2A			MEF2A
MEF2B	MEF2B	MEF2B	MEF2B
MEN1	MEN1	MEN1	MEN1
MET	MET	MET	MET
MGA	MGA	MGA	MGA
MGMT		MGMT	
MITF	MITF	MITF	MITF
MLH1	MLH1	MLH1	MLH1
MLH3	MLH3	MLH3	MLH3
MLLT10	MLLT10	MLLT10	MLLT10
MLLT3	MLLT3	MLLT3	MLLT3
MMP2	MMP2		MMP2
MN1	MN1	MN1	MN1
MNDA			MNDA
MOB1A	MOB1A		
MOB1B	MOB1B		
MPL	MPL	MPL	MPL
MPO	MPO		MPO
MRE11A	MRE11A	MRE11A	MRE11A
MS4A1		MS4A1	
MSH2	MSH2	MSH2	MSH2
MSH3	MSH3	MSH3	
MSH4		MSH4	
MSH5		MSH5	
MSH6	MSH6	MSH6	MSH6
MSR1	MSR1	MSR1	
MST1R		MST1R	
MTHFR	MTHFR	MTHFR	MTHFR
MTOR	MTOR	MTOR	MTOR
MTR	MTR		
MTRR	MTRR	MTRR	
MUC1	MUC1	MUC1	
MUC16		MUC16	
MUTYH	MUTYH	MUTYH	MUTYH
MXI1	MXI1	MXI1	
MYB	MYB	MYB	MYB
MYBPC3			MYBPC3
MYC	MYC	MYC	MYC
MYCL	MYCL	MYCL	MYCL

MYCN	MYCN	MYCN	MYCN
MYD88	MYD88	MYD88	MYD88
MYH11	MYH11	MYH11	
MYH7			MYH7
MYH9	MYH9	MYH9	MYH9
MYL2			MYL2
MYL3			MYL3
MYLK			MYLK
MYOD1			MYOD1
NAT1	NAT1		
NAT2	NAT2		
NAV3			NAV3
NBN	NBN	NBN	NBN
NCOA1	NCOA1	NCOA1	
NCOA2	NCOA2		
NCOA3	NCOA3	NCOA3	NCOA3
NCOR1	NCOR1	NCOR1	NCOR1
NCOR2			NCOR2
NEDD4L			NEDD4L
NF1	NF1	NF1	NF1
NF2	NF2	NF2	NF2
NFE2L2	NFE2L2	NFE2L2	NFE2L2
NFKB1	NFKB1	NFKB1	NFKB1
NFKB2	NFKB2	NFKB2	NFKB2
NFKBIA	NFKBIA	NFKBIA	NFKBIA
NFKBIE		NFKBIE	
NIN	NIN	NIN	NIN
NIPBL			NIPBL
NKX2-1	NKX2-1		NKX2-1
NKX3-1	NKX3-1		
NLRC5		NLRC5	
NOP10		NOP10	
NOTCH1	NOTCH1	NOTCH1	NOTCH1
NOTCH2	NOTCH2	NOTCH2	NOTCH2
NOTCH3	NOTCH3	NOTCH3	NOTCH3
NOTCH4		NOTCH4	NOTCH4
NPM1	NPM1	NPM1	NPM1
NQO1		NQO1	NQO1
NR1I2			
NR1I3		NR1I3	
NR4A2			NR4A2
NRAS	NRAS	NRAS	NRAS
NRG2		NRG2	
NSD1	NSD1	NSD1	NSD1
NT5C2		NT5C2	
NTHL1		NTHL1	
NTN4			NTN4
NTRK1	NTRK1	NTRK1	NTRK1
NTRK2	NTRK2	NTRK2	NTRK2
NTRK3	NTRK3	NTRK3	NTRK3

NUMA1	NUMA1	NUMA1	NUMA1
NUP93	NUP93		NUP93
NUP98	NUP98	NUP98	NUP98
NUTM1			NUTM1
OR5A1			OR5A1
OTC			OTC
P2RY1			
P2RY12			
PABPC1			PABPC1
PAK1			PAK1
PAK3	PAK3	PAK3	PAK3
PAK7			PAK7
PALB2	PALB2	PALB2	PALB2
PALLD	PALLD	PALLD	
PARK2	PARK2	PARK2	PARK2
PARP1		PARP1	PARP1
PARP2		PARP2	PARP2
PARP3			PARP3
PARP4		PARP4	PARP4
PAX3	PAX3	PAX3	
PAX5	PAX5	PAX5	PAX5
PAX7	PAX7	PAX7	
PAX8			PAX8
PBK		PBK	
PBRM1	PBRM1	PBRM1	PBRM1
PBX1	PBX1	PBX1	
PCBP1	PCBP1		PCBP1
PCSK9			PCSK9
PDCD1		PDCD1	PDCD1
PDCD1LG2	PDCD1LG2	PDCD1LG2	
PDF		PDF	
PDGFA		PDGFA	
PDGFB	PDGFB	PDGFB	
PDGFC		PDGFC	
PDGFD		PDGFD	
PDGFRA	PDGFRA	PDGFRA	PDGFRA
PDGFRB	PDGFRB	PDGFRB	PDGFRB
PDK1	PDK1	PDK1	PDK1
PDPK1			PDPK1
PER1	PER1		
PGR		PGR	
PHF6	PHF6	PHF6	PHF6
PHLPP2			
PHOX2B	PHOX2B	PHOX2B	PHOX2B
PIAS4		PIAS4	
PIGA		PIGA	
PIK3C2A		PIK3C2A	
PIK3C2B	PIK3C2B	PIK3C2B	PIK3C2B
PIK3C2G		PIK3C2G	PIK3C2G
PIK3C3			PIK3C3

PIK3CA	PIK3CA	PIK3CA	PIK3CA
PIK3CB	PIK3CB	PIK3CB	PIK3CB
PIK3CD	PIK3CD	PIK3CD	PIK3CD
PIK3CG	PIK3CG	PIK3CG	PIK3CG
PIK3R1	PIK3R1	PIK3R1	PIK3R1
PIK3R2	PIK3R2	PIK3R2	PIK3R2
PIK3R3		PIK3R3	
PIM1	PIM1	PIM1	PIM1
PIP5K1A			PIP5K1A
PKHD1	PKHD1	PKHD1	
PKP2			PKP2
PLCG1	PLCG1	PLCG1	PLCG1
PLCG2	PLCG2	PLCG2	PLCG2
PLK2			PLK2
PMAIP1			PMAIP1
PML	PML	PML	PML
PMS1	PMS1	PMS1	PMS1
PMS2	PMS2	PMS2	PMS2
PNRC1			PNRC1
POLD1	POLD1	POLD1	POLD1
POLE	POLE	POLE	POLE
POLH	POLH	POLH	
POLQ	POLQ	POLQ	POLQ
POLR3B			POLR3B
POT1	POT1	POT1	
POU2AF1	POU2AF1		
POU2F2	POU2F2		POU2F2
POU5F1	POU5F1		
PPM1D	PPM1D	PPM1D	PPM1D
PPP2R1A	PPP2R1A		PPP2R1A
PPP6C			PPP6C
PRAM1			
PREX2			
PRDM1	PRDM1	PRDM1	PRDM1
PRDM16	PRDM16	PRDM16	
PREX2	PREX2	PREX2	
PRF1	PRF1	PRF1	
PRKACA	PRKACA		
PRKAG2			PRKAG2
PRKAR1A	PRKAR1A	PRKAR1A	PRKAR1A
PRKCA		PRKCA	
PRKCI	PRKCI		
PRKD1	PRKD1	PRKD1	
PRKDC	PRKDC	PRKDC	PRKDC
PROM2		PROM2	
PRPF8			PRPF8
PRSS1	PRSS1	PRSS1	
PRSS8	PRSS8		PRSS8
PRX	PRX	PRX	PRX
PSIP1	PSIP1	PSIP1	PSIP1

PSMB1		PSMB1	
PSMB10		PSMB10	
PSMB2		PSMB2	
PSMB5		PSMB5	
PSMB8		PSMB8	
PSMB9		PSMB9	
PSMC3IP		PSMC3IP	
PSPH	PSPH	PSPH	
PTCH1	PTCH1	PTCH1	PTCH1
PTCH2		PTCH2	
PTEN	PTEN	PTEN	PTEN
PTGIS			
PTGS2	PTGS2	PTGS2	PTGS2
PTK2		PTK2	
PTK7		PTK7	
PTPN11	PTPN11	PTPN11	PTPN11
PTPRC	PTPRC	PTPRC	PTPRC
PTPRD	PTPRD	PTPRD	PTPRD
PTPRS			PTPRS
PTPRT	PTPRT	PTPRT	PTPRT
QKI	QKI		QKI
RAC1	RAC1	RAC1	RAC1
RAC2		RAC2	
RAD21	RAD21	RAD21	RAD21
RAD50	RAD50	RAD50	RAD50
RAD51	RAD51	RAD51	RAD51
RAD51B	RAD51B	RAD51B	RAD51B
RAD51C	RAD51C	RAD51C	RAD51C
RAD51D	RAD51D	RAD51D	RAD51D
RAD52			RAD52
RAD54B		RAD54B	
RAD54L		RAD54L	RAD54L
RAF1	RAF1	RAF1	RAF1
RALGDS	RALGDS	RALGDS	
RANBP2			RANBP2
RARA	RARA	RARA	RARA
RARB		RARB	
RARG		RARG	
RASA1	RASA1	RASA1	RASA1
RASAL1	RASAL1	RASAL1	
RB1	RB1	RB1	RB1
RBM10	RBM10	RBM10	RBM10
RBM15	RBM15		
RBMX			RBMX
RECQL	RECQL		
RECQL4	RECQL4	RECQL4	RECQL4
REL	REL	REL	REL
RET	RET	RET	RET
RFC2		RFC2	
RFWD2			RFWD2

RFX5		RFX5	
RGL1			
RGL2			
RHBDF2		RHBDF2	RHBDF2
RHEB	RHEB	RHEB	RHEB
RHOA	RHOA	RHOA	RHOA
RHOH	RHOH		
RICTOR	RICTOR	RICTOR	RICTOR
RINT1	RINT1	RINT1	
RIPK1		RIPK1	
RIT1		RIT1	RIT1
RNASEL	RNASEL	RNASEL	
RNF2	RNF2	RNF2	
RNF43	RNF43	RNF43	RNF43
ROS1	ROS1	ROS1	ROS1
RPA1			RPA1
RPGR			RPGR
RPL22	RPL22	RPL22	RPL22
RPL5	RPL5		RPL5
RPS15			RPS15
RPS20		RPS20	
RPS6KA4			RPS6KA4
RPS6KB1		RPS6KB1	
RPS6KB2			RPS6KB2
RPTOR	RPTOR	RPTOR	RPTOR
RRM1	RRM1		
RSF1		RSF1	
RUNX1	RUNX1	RUNX1	RUNX1
RUNX1T1	RUNX1T1		RUNX1T1
RUNX3			RUNX3
RXRA	RXRA		RXRA
RYBP			RYBP
RYR1	RYR1	RYR1	RYR1
SACS	SACS	SACS	SACS
SALL4			
SAMHD1		SAMHD1	
SAV1	SAV1	SAV1	SAV1
SBDS	SBDS	SBDS	SBDS
SCG5		SCG5	
SCN11A			SCN11A
SCN5A			SCN5A
SDHA	SDHA	SDHA	SDHA
SDHAF2	SDHAF2	SDHAF2	SDHAF2
SDHB	SDHB	SDHB	SDHB
SDHC	SDHC	SDHC	SDHC
SDHD	SDHD	SDHD	SDHD
SEC23B		SEC23B	
SELP	SELP		
SEMA4A	SEMA4A	SEMA4A	
SEPT9	SEPT9		

SETBP1	SETBP1	SETBP1	SETBP1
SETD2	SETD2	SETD2	SETD2
SETDB1	SETDB1	SETDB1	SETDB1
SF3B1	SF3B1	SF3B1	SF3B1
SGK1	SGK1	SGK1	SGK1
SH2B1		SH2B1	
SH2B3		SH2B3	SH2B3
SH2D1A	SH2D1A	SH2D1A	SH2D1A
SHFM1		SHFM1	
SHH		SHH	
SHQ1			SHQ1
SIK2		SIK2	
SIN3A	SIN3A	SIN3A	SIN3A
SIRT1		SIRT1	
SKP2	SKP2	SKP2	
SLC15A2	SLC15A2		
SLC19A1			SLC19A1
SLC1A3	SLC1A3		
SLC22A1	SLC22A1		
SLC22A2	SLC22A2		SLC22A2
SLC22A6	SLC22A6		
SLC26A3	SLC26A3	SLC26A3	SLC26A3
SLCO1B1	SLCO1B1		
SLCO1B3	SLCO1B3		SLCO1B3
SLIT2	SLIT2	SLIT2	
SLX4	SLX4	SLX4	
SMAD2	SMAD2		SMAD2
SMAD3	SMAD3	SMAD3	SMAD3
SMAD4	SMAD4	SMAD4	SMAD4
SMARCA1			SMARCA1
SMARCA4	SMARCA4	SMARCA4	SMARCA4
SMARCB1	SMARCB1	SMARCB1	SMARCB1
SMARCD1			SMARCD1
SMARCE1	SMARCE1	SMARCE1	
SMC1A	SMC1A	SMC1A	SMC1A
SMC3	SMC3	SMC3	SMC3
SMO	SMO	SMO	SMO
SMUG1	SMUG1		
SNCAIP	SNCAIP		SNCAIP
SOCS1	SOCS1	SOCS1	SOCS1
SOD2			SOD2
SOS1	SOS1		SOS1
SOX10	SOX10		SOX10
SOX11	SOX11	SOX11	
SOX17	SOX17		SOX17
SOX2	SOX2	SOX2	SOX2
SOX9	SOX9	SOX9	SOX9
SPEN	SPEN	SPEN	SPEN
SPINK1	SPINK1	SPINK1	
SPOP	SPOP	SPOP	SPOP

SPOPL			
SPRED1	SPRED1	SPRED1	
SPTA1	SPTA1	SPTA1	SPTA1
SPTAN1			SPTAN1
SRC	SRC	SRC	SRC
SRD5A2	SRD5A2	SRD5A2	
SRGAP1		SRGAP1	
SRP72		SRP72	
SRSF2	SRSF2	SRSF2	SRSF2
SSTR1		SSTR1	
SSTR2		SSTR2	
SSTR3		SSTR3	
SSTR5		SSTR5	
SSX1	SSX1	SSX1	
STAG1	STAG2	STAG1	
STAG2		STAG2	STAG2
STAT1		STAT1	
STAT3	STAT3	STAT3	STAT3
STAT4	STAT4		STAT4
STAT5A		STAT5A	
STAT5B	STAT5B	STAT5B	
STK11	STK11	STK11	STK11
STK19			
STK3	STK3		
STK31			
STK4	STK4		
STK40			STK40
SUFU	SUFU	SUFU	SUFU
SULT1A1	SULT1A1		SULT1A1
SUZ12	SUZ12	SUZ12	SUZ12
SYK	SYK	SYK	SYK
SYNE1			SYNE1
TAF1	TAF1	TAF1	TAF1
TAF15	TAF15	TAF15	
TAL1	TAL1		
TAP1	TAP1	TAP1	TAP1
TAP2		TAP2	
TBK1		TBK1	
TBL1XR1	TBL1XR1	TBL1XR1	TBL1XR1
TBX22			
TBX3	TBX3	TBX3	TBX3
TCF12			TCF12
TCF3	TCF3	TCF3	TCF3
TCF7L1	TCF7L1		
TCF7L2	TCF7L2	TCF7L2	TCF7L2
TCL1A	TCL1A	TCL1A	
TEK		TEK	
TERC	TERC	TERC	
TERF2IP	TERF2IP	TERF2IP	
TERT	TERT	TERT	TERT

TET1	TET1	TET1	TET1
TET2	TET2	TET2	TET2
TFDP1			TFDP1
TFE3	TFE3	TFE3	
TGFBR1			TGFBR1
TGFBR2	TGFBR2	TGFBR2	TGFBR2
TGIF1			TGIF1
THBS1	THBS1		
TIMP3	TIMP3		
TIPARP			TIPARP
TJP2			TJP2
TLR4	TLR4	TLR4	TLR4
TLX1	TLX1	TLX1	
TLX3	TLX3		
TMEM127	TMEM127	TMEM127	TMEM127
TMEM43			TMEM43
TMPRSS2	TMPRSS2		TMPRSS2
TNF	TNF	TNF	TNF
TNFAIP3	TNFAIP3	TNFAIP3	TNFAIP3
TNFRSF11A		TNFRSF11A	
TNFRSF13B		TNFRSF13B	
TNFRSF14	TNFRSF14	TNFRSF14	TNFRSF14
TNFRSF1A		TNFRSF1A	
TNFRSF1B		TNFRSF1B	
TNFRSF25		TNFRSF25	
TNFRSF8		TNFRSF8	
TNFSF11		TNFSF11	
TNK2	TNK2	TNK2	
TNKS			
TNKS2			
TNNI3			TNNI3
TNNT2			TNNT2
TNPO1			TNPO1
TOM1			TOM1
TOP1	TOP1	TOP1	TOP1
TOP2A	TOP2A	TOP2A	TOP2A
TP53	TP53	TP53	TP53
TP53BP1	TP53BP1	TP53BP1	TP53BP1
TP63			TP63
TPMT	TPMT		TPMT
TPX2	TPX2	TPX2	TPX2
TRAF2		TRAF2	
TRAF3	TRAF3	TRAF3	TRAF3
TRAF5		TRAF5	
TRAF6		TRAF6	
TRAF7	TRAF7	TRAF7	TRAF7
TRIM24	TRIM24		
TRIM28			
TRIO			TRIO
TRRAP	TRRAP	TRRAP	TRRAP

TSC1	TSC1	TSC1	TSC1
TSC2	TSC2	TSC2	TSC2
TSHR	TSHR	TSHR	TSHR
TTK			TTK
TUBA4A		TUBA4A	
TUBB		TUBB	
TXNIP			TXNIP
TYMS	TYMS	TYMS	TYMS
U2AF1	U2AF1	U2AF1	U2AF1
UBE2T		UBE2T	
UBR5	UBR5	UBR5	UBR5
UGT1A1	UGT1A1		UGT1A1
UGT2B15	UGT2B15	UGT2B15	
UGT2B17	UGT2B17		
UGT2B7	UGT2B7	UGT2B7	
UIMC1	UIMC1	UIMC1	
UNG		UNG	
UPF3B			UPF3B
USP34		USP34	
USP9X	USP9X	USP9X	USP9X
VDR			
VEGFA	VEGFA	VEGFA	
VEGFB		VEGFB	
VHL	VHL	VHL	VHL
VKORC1	VKORC1	VKORC1	
VTCN1			VTCN1
WAS		WAS	
WASF3	WASF3	WASF3	WASF3
WHSC1	WHSC1	WHSC1	WHSC1
WHSC1L1			WHSC1L1
WISP3	WISP3	WISP3	WISP3
WNK1			WNK1
WRN	WRN	WRN	WRN
WT1	WT1	WT1	WT1
WWTR1	WWTR1		
XIAP		XIAP	XIAP
XPA	XPA	XPA	XPA
XPC	XPC	XPC	XPC
XPO1	XPO1	XPO1	XPO1
XRCC1	XRCC1	XRCC1	
XRCC2	XRCC2	XRCC2	XRCC2
XRCC3		XRCC3	XRCC3
XRCC5		XRCC5	
XRCC6		XRCC6	
YAP1	YAP1	YAP1	YAP1
YES1			YES1
ZBTB2	ZBTB2		
ZFHX3	ZFHX3	ZFHX3	ZFHX3
ZFP36L1			ZFP36L1
ZFP36L2			ZFP36L2

ZHX3		ZHX3	
ZMYM2			ZMYM2
ZMYM3			ZMYM3
ZNF217	ZNF217	ZNF217	ZNF217
ZNF703			ZNF703
ZNF750			ZNF750
ZNF814			ZNF814
ZNRF3		ZNRF3	
ZNF703	ZNF703		
ZRSR2		ZRSR2	ZRSR2
