# Supplementary figures and images for: Clinical and Genetic Tumor Characteristics of Responding and Non-Responding Patients to PD-1 Inhibition in Hepatocellular Carcinoma
Source: Cancers (Basel). 2020 Dec 18;12(12):3830. doi: 10.3390/cancers12123830 (PMC7766321; doi:10.3390/cancers12123830)

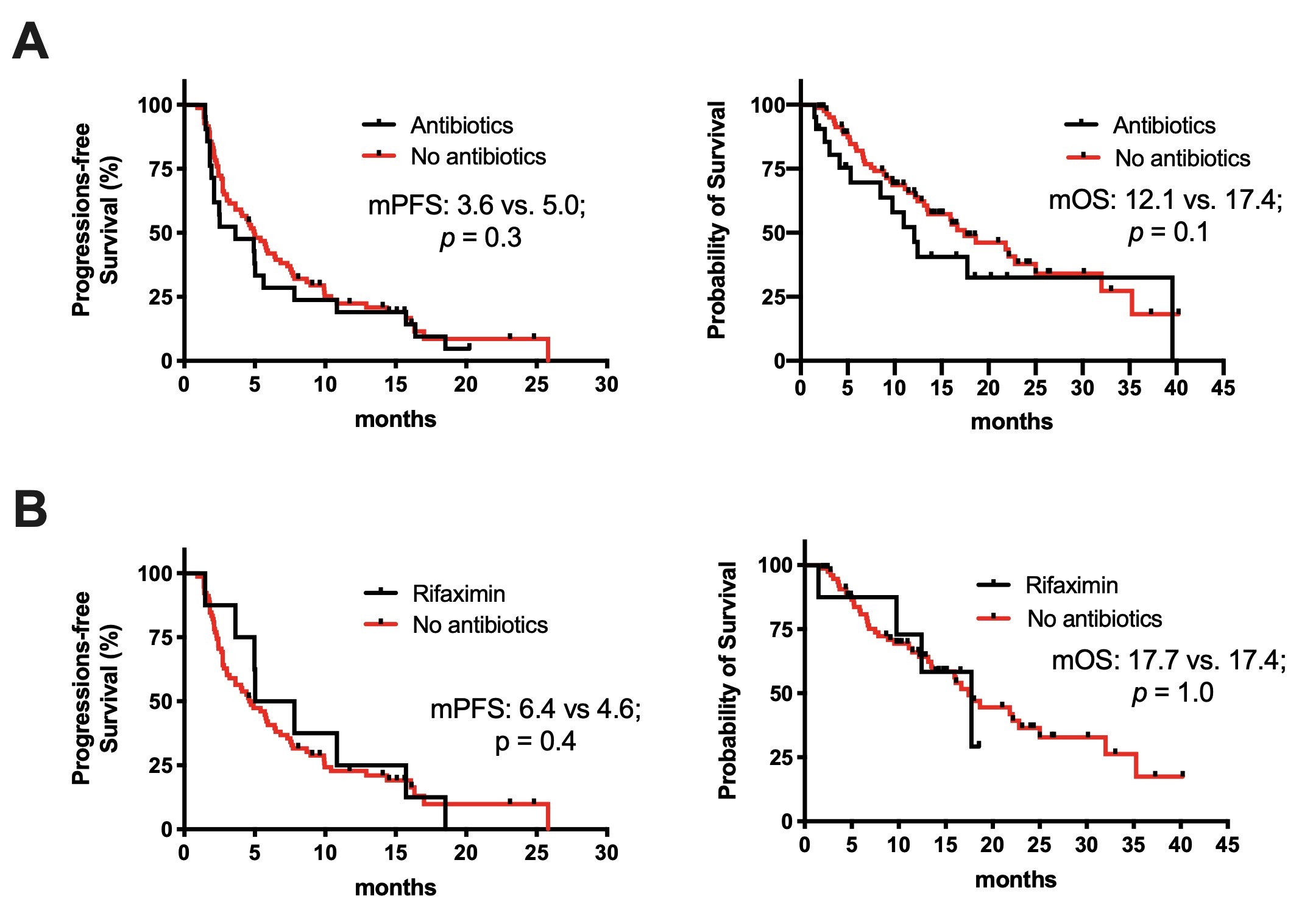

Supplement: Supplementary file 1 [file cancers-12-03830-s001.zip › supp/Figure S1_colour_image.jpg]
